# Reappraisal of the Subtropical Guidelines on Palivizumab Prophylaxis in Congenital Heart Disease

**DOI:** 10.3389/fped.2021.756787

**Published:** 2022-01-05

**Authors:** Shuenn-Nan Chiu, Ching-Chia Wang, Ming-Tai Lin, Chun-An Chen, Chun-Wei Lu, Yu-Chuan Hua, Jing-Ming Wu, Mei-Hwan Wu, Jou-Kou Wang

**Affiliations:** ^1^Department of Pediatrics, National Taiwan University Hospital, Taipei, Taiwan; ^2^Department of Pediatrics, Medical College, National Taiwan University, Taipei, Taiwan; ^3^Cardiac Children's Foundation Taiwan, Taipei, Taiwan; ^4^Department of Pediatrics, National Cheng Kung University Hospital, Tainan, Taiwan

**Keywords:** palivizumab prophylaxis, respiratory syncytial virus, congenital heart disease, genetic syndrome, lung abnormality, airway abnormality

## Abstract

**Objective:** To define the impact of associated abnormalities on the efficacy of the novel subtropical guidelines for palivizumab prophylaxis on respiratory syncytial virus (RSV)-related hospitalizations in patients with hemodynamically significant congenital heart disease (hsCHD).

**Method:** This prospective study enrolled every patient seen at a tertiary care center for hsCHD, who was born between 2014 and 2018 and received at least 1 dose of palivizumab, according to the subtropical guidelines. The patients were followed until the age of 2 years.

**Results:** A total of 772 patients (49% male) were enrolled. Cyanotic CHD was seen in 46% of patients, of whom 23% had associated abnormalities. Lung/airway abnormalities (14%) were the most common followed by the genetic syndromes associated with CHD (7.3%). Among the 772 patients, RSV-related hospitalizations occurred in 3.2 and 2.2% children aged ≤ 12 and 13–24 months, respectively. Most of the RSV infections occurred in patients no longer satisfying the criteria for palivizumab prophylaxis. The patients with associated abnormalities but not the type of CHD, patient age, and patient sex were risk factors for RSV-related hospitalizations. The rates of RSV-related hospitalizations, admission to the intensive care unit, and endotracheal intubation were higher for patients with associated anomalies than for other patients before 24 months of age (10.2 vs. 4.0%, 67 vs. 33%, and 39 vs. 4.2%, *p* = 0.004, 0.06, 0.013, respectively).

**Conclusion:** Children with abnormalities, especially genetic syndromes and lung/airway problems associated with CHD, are at high risk for RSV-related hospitalization. Our current subtropical guidelines for palivizumab prophylaxis in patients with hsCHD, should be revised to include the results of this study.

## Introduction

Respiratory syncytial virus (RSV) is the most common cause of admission for lower respiratory tract infections in children aged younger than 2 years (1, 2). The hospitalization rate is especially high in patients with underlying conditions such as congenital heart disease (CHD), prematurity, chronic lung disease, and immunodeficiency. The hospitalization rate and need for intensive care and ventilator support are also higher in children with CHD than in children without underlying diseases (3–8). Monthly injections of the humanized monoclonal antibody palivizumab have been shown to reduce the rates of RSV-related hospitalization and severity of RSV infection (4, 9).

The American Academy of Pediatrics (AAP) has recommended a monthly injection of palivizumab for all patients with hemodynamically significant acyanotic CHD and for some patients with cyanotic CHD for total 5 shots during the season when RSV is prevalent (10). In a nationwide study, we found that that those with cyanotic CHD had higher RSV-related hospitalization rates and more severe disease than those with acyanotic CHD (11). We proposed a novel protocol for the use of palivizumab to prevent RSV infection in children with CHD who live in subtropical regions where RSV infections occur throughout the year and are not seasonally related (12). In brief, all patients with cyanotic CHD and hemodynamically significant (hs) acyanotic CHD aged younger than 1 year should receive monthly palivizumab prophylaxis for a maximum of 6 doses after the diagnosis of CHD if they fulfilled the criteria of hsCHD. The efficacy of this subtropical prophylaxis protocol has been validated by a multicenter study of patients living in a subtropical region (12).

Although palivizumab prophylaxis for patients with CHD as based on this subtropical guideline has been effective, there remain patients who develop RSV infections. CHD is well-known to be associated with comorbidities such as genetic disorders, airway abnormalities, neurological abnormalities, and multiple anomalies (13). Whether or not these comorbidities affect the occurrence of RSV infections in patients treated according to the current guidelines for the use of palivizumab prophylaxis remains unknown. In this study, we determined the rate of RSV-related hospitalization in patients aged < 1 year with cyanotic and acyanotic hemodynamically significant CHD (hsCHD) who received palivizumab prophylaxis in accordance with the subtropical guidelines. We also investigated the causes of hospitalization and risk factors for RSV infections in patients treated based on the subtropical guidelines.

## Methods

### Subjects

This is a prospective single center observational study. All patients with hsCHD who were born between Jan 2014 and Dec 2018 and received at least 1 dose of palivizumab at National Taiwan University Hospital were enrolled in our study and followed until the age of 2 years. Data were collected in accordance with the policy of the institutional review board at our hospital and the reference number was 201307004RINC. The study was carried out in accordance with The Code of Ethics of the World Medical Association for experiments involving humans. The informed consent forms were signed by their parents.

The sputum or nasopharyngeal samples of all patients hospitalized for respiratory tract infections before the ages of 2 years were routinely tested for RSV antigen and/or cultured for viruses. The patients' clinical characteristics and history of previous hospitalization for RSV-associated respiratory tract infections were also recorded.

### Subtropical Palivizumab Prophylaxis Protocol

The palivizumab prophylaxis protocol consisted of maximal 6 injections (injection dose: 15 mg palivizumab/kg) at intervals of at least 4 weeks following the diagnosis of hsCHD. Patients were required to be younger than 1 year of age, and to satisfy the criteria for hsCHD at each time of each injection. This requirement ensured that if the cardiac condition of the patient improved, the patient would not receive palivizumab.

The definitions of hsCHD were as follows: (1) cyanotic CHD before total correction (through surgery or transcatheter intervention) or after total correction but with residual cyanosis or signs/symptoms of heart failure; or (2) acyanotic CHD with signs/symptoms of heart failure, either before or after total correction. The presence of signs/symptoms of heart failure was based on satisfying at least 2 of the following 3 criteria: (1) failure to thrive, with a body weight lower than the third percentile; (2) significant cardiomegaly (assessed through imaging studies); and (3) at least 2 medications needed to control heart failure.

### Additional Definitions

“Very preterm” was defined as birth occurring at a gestational age of ≤32 weeks. “Associated abnormalities” were defined as major congenital anomalies and acquired abnormalities requiring further management, which included but were not restricted to the following: patients with a “genetic syndrome” such as Down syndrome, patients with multiple anomalies, or patients with a specific syndrome such as the VACTERAL syndrome. “Lung/airway anomaly” included the following: (1) major lung abnormality including congenital anomaly or an acquired lesion such as diaphragmatic palsy; (2) tracheal or bronchial stenosis or tracheomalacia or bronchomalacia requiring evaluation by flexible bronchoscopy; (3) “neurological abnormalities” included the following: congenital brain malformations or acquired lesions such as intracranial hemorrhage or hypoxic encephalopathy due to either injuries associated with birth or later surgical procedures.

### Data Analysis

Data are expressed as means ± standard deviation. The Student *t*-test was used for numerical data, and the Chi-squared or Fisher exact test was used for categorical data. Differences were deemed significant for *p* < 0.05.

## Results

### Basic Demographic Data

A total of 772 patients (49% male) were enrolled. Basic demographic data are shown in Table 1. Cyanotic and acyanotic CHD were seen in 46 and 54%, respectively, of the study patients. The proportions of patients who were premature with a gestational age younger than 32 weeks, or had associated abnormalities were 3 and 23%, respectively. Lung/airway abnormalities were the most common associated abnormalities, followed by genetic syndromes. The mean number of palivizumab injections received by the patients was 3.3 (Supplementary Table 1). Supplementary Table 2 shows the numbers of palivizumab injections administered to patients stratified according to different associated abnormalities. No serious adverse event was found after palivizumab injection.

**Table 1:** Basic demographics of patients with hemodynamically significant congenital heart disease (CHD) (*n* = 772).

**Total**	**All**	**RSV-positive**	**Not-hospitalized**
		**hospitalization**	**(RSV-negative)**
Number	772	42	730
Gender (male, %)	379 (49%)	21 (50%)	358 (49%)
Cyanotic CHD (%)	358 (46%)	18 (43%)	340 (47%)
Premature (%)	23 (3%)	2 (4.8%)	21 (2.9%)
Gestational age	37.7 ± 2.2	37.6 ± 2.3	37.7 ± 2.2
Birth weight (kilogram)	2.8 ± 0.6	2.8 ± 0.7	2.8 ± 0.6
Previous surgery	544 (70.5%)	33 (78.6%)	511 (70%)
Associated abnormalities^*^	177 (23%)	18 (43%)	159 (22%)
Genetic syndromes (%)^**^	56 (7.3%)	7 (16.7%)	49 (6.7%)
Lung/airway abnormalities^*^	104 (14%)	13 (31%)	91 (13%)
Neurologic abnormalities	44 (5.7%)	4 (9.5%)	40 (5.5%)

* *denotes p < 0.01*,

** *denotes p < 0.05*.

### RSV-Related Hospitalization

A total of 42 patients were hospitalized a total of 43 times for RSV infections (1 patient was hospitalized twice). The 2-year RSV-related hospitalization rate was 5.5%. The hospitalization rate was 3.2% for patients aged ≤ 12 months and 2.2% for patients aged 13–24 months (*p* = 0.21). The mean duration of hospitalization was 10 ± 8.5 days. No patient died of RSV infection in this cohort.

The 2-year hospitalization rates of patients with cyanotic (5.0%) vs. acyanotic (5.8%) CHD were similar (*p* = 0.64). The associations between RSV-related hospitalizations and palivizumab prophylaxis are shown in Supplementary Figure 1. Less than 10% of RSV-related hospitalization occurred in patients during the palivizumab prophylaxis period. Most of the infections occurred in patients (69%) who no longer satisfied the criteria for palivizumab prophylaxis and in patients (17%) before palivizumab prophylaxis was initiated because the diagnosis of CHD was delayed.

**Figure 1:**
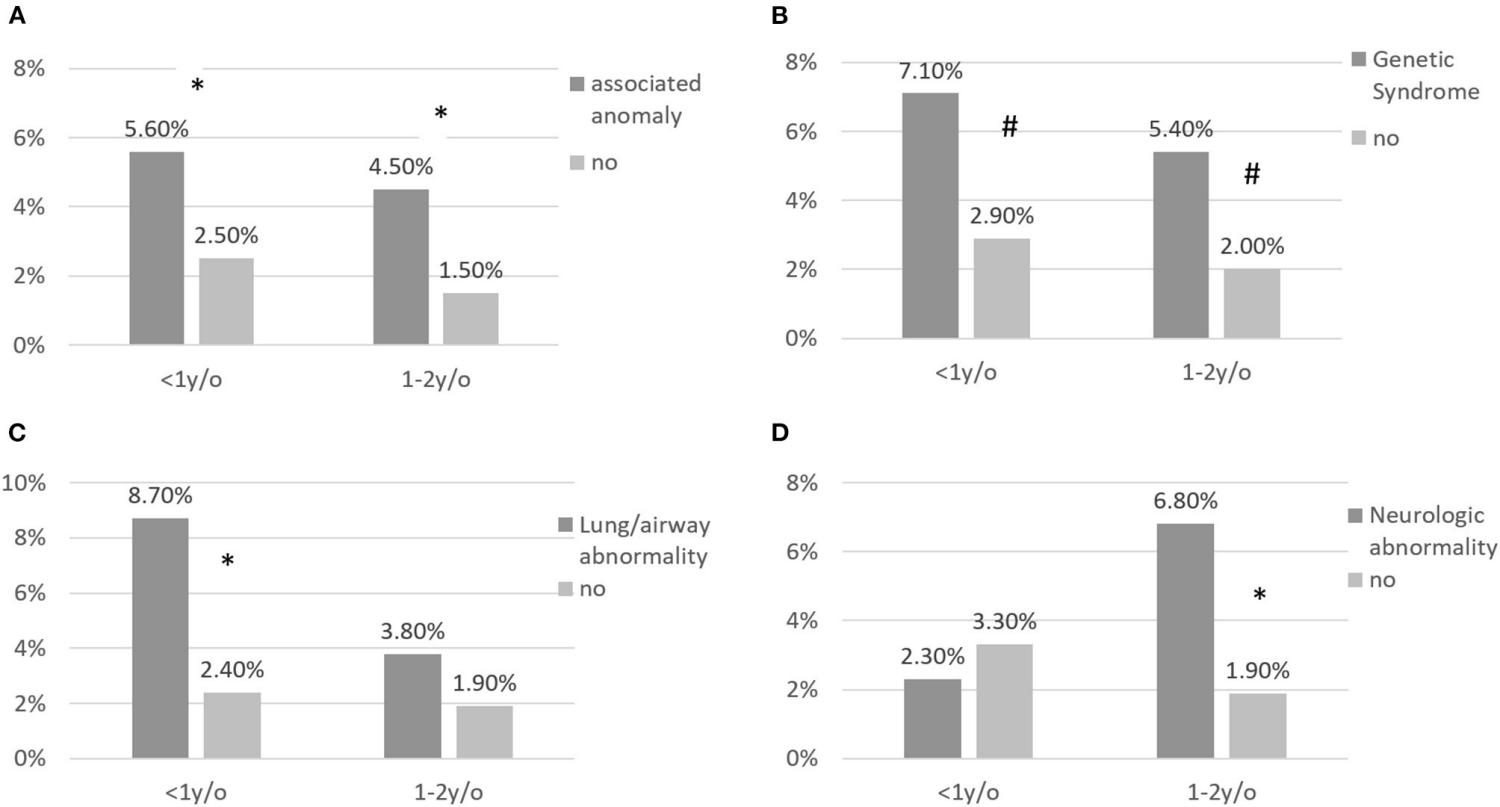
**(A–D)** RSV-related hospitalization for children with CHD according to associated abnormalities and stratified by age at infection. RSV-related hospitalization rates were significantly higher for patients with associated abnormalities aged < 1 and aged >1 and <2 years. * annotates *p* < 0.05; # annotates *p* < 0.1.

The impacts of associated anomalies on the rates of RSV-related hospitalizations for different age groups are shown in Figure 1. For children with associated anomalies aged ≤ 12 months and those aged 13–24 months, the RSV-related hospitalization rates were 5.6 and 4.5%, respectively, which were significantly higher than for children without associated anomalies aged ≤ 12 months (2.5%, *p* = 0.04) and those aged 13–24 months (1.5%, *p* = 0.016). Children with genetic syndromes and those with lung/airway abnormalities also showed higher RSV-related hospitalization rates than children without genetic syndromes or lung/airway abnormalities.

### Intensive Care Unit Admission and Intubation Rate

Among the patients requiring admission for RSV infections, the rates of ICU admission and endotracheal intubation were 47.6 and 19%, respectively. The mean duration of ICU stay was 10.7 ± 8.1 days. None of the differences between the rates of ICU admission and endotracheal intubation for patients with acyanotic CHD vs. those with cyanotic CHD were significant: the rates for ICU admission were 46 and 50%, respectively (*p* = 1.00), and for endotracheal intubation were 25 and 11%, respectively (*p* = 0.43). None of the differences between the rates of ICU admission and endotracheal intubation were significant for patients aged ≤ 12 months and those aged 13–24 months: the rates for ICU admission were 56 and 35%, respectively (*p* = 0.22), and for endotracheal intubation were 24 and 12%, respectively (*p* = 0.44).

**Figure 2:**
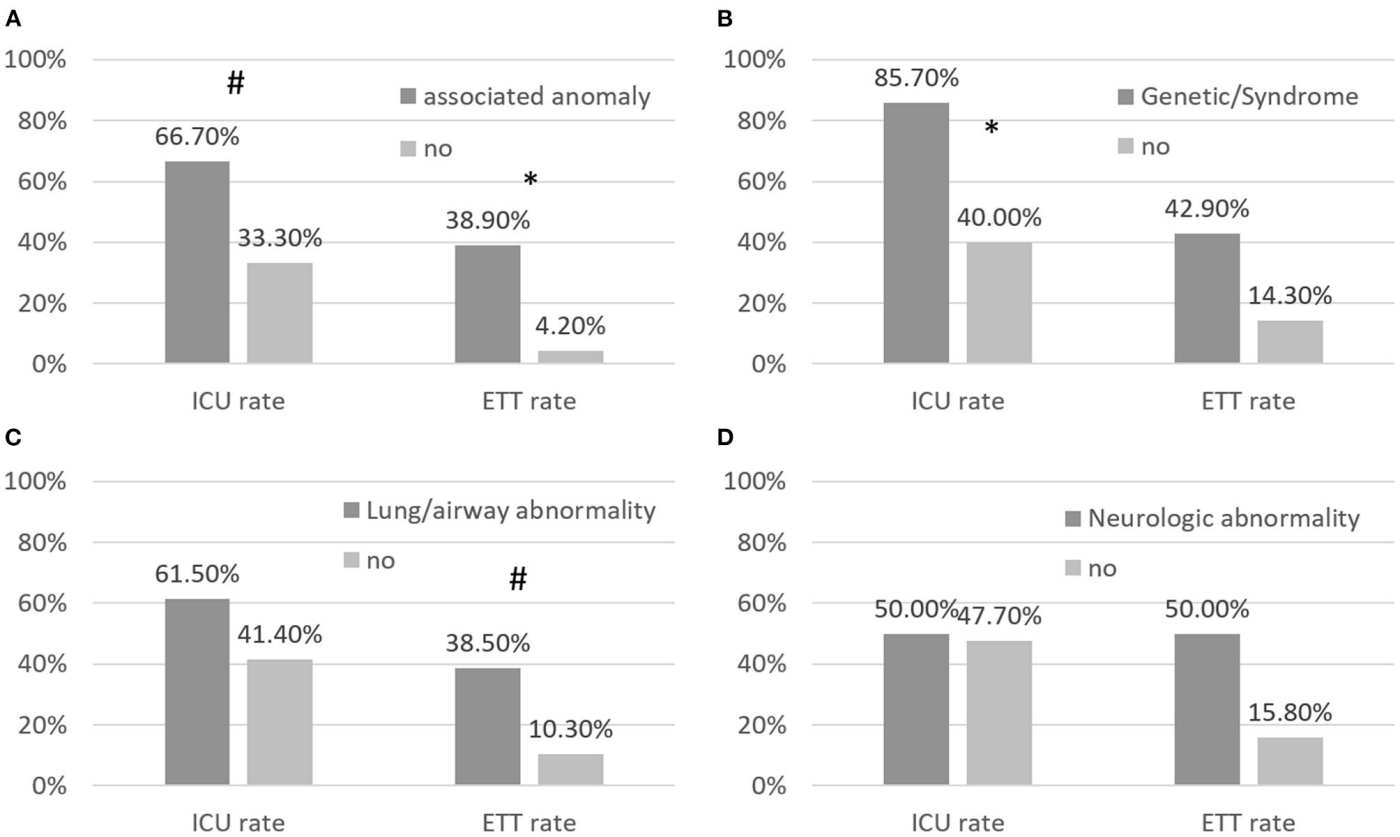
**(A–D)** Rates of RSV-related admission to the intensive care unit (ICU) and rates of endotracheal intubation (ETT) in children with CHD and associated abnormalities. Patients with associated abnormalities had borderline higher RSV-related rates of ICU admission and ETT than patients with CHD without associated abnormalities. * annotates *p* < 0.05; # annotates *p* < 0.1.

Figure 2 shows the rates of ICU admission and endotracheal intubation of patients with different associated anomalies. The ICU admission rate was 67% in patients with and 33% in patients without associated anomalies (*p* = 0.06). The endotracheal intubation rate was 39% in patients with and 4.2% in patients without associated anomalies (*p* = 0.013). The ICU admission rate was significantly higher in patients with than in patients without genetic syndromes, and the endotracheal intubation rate was borderline higher in patients with than in patients without lung/airway abnormalities.

### Ages of Patients Needing Hospitalization for RSV Infections and Seasonality of RSV Infections

**Figure 3:**
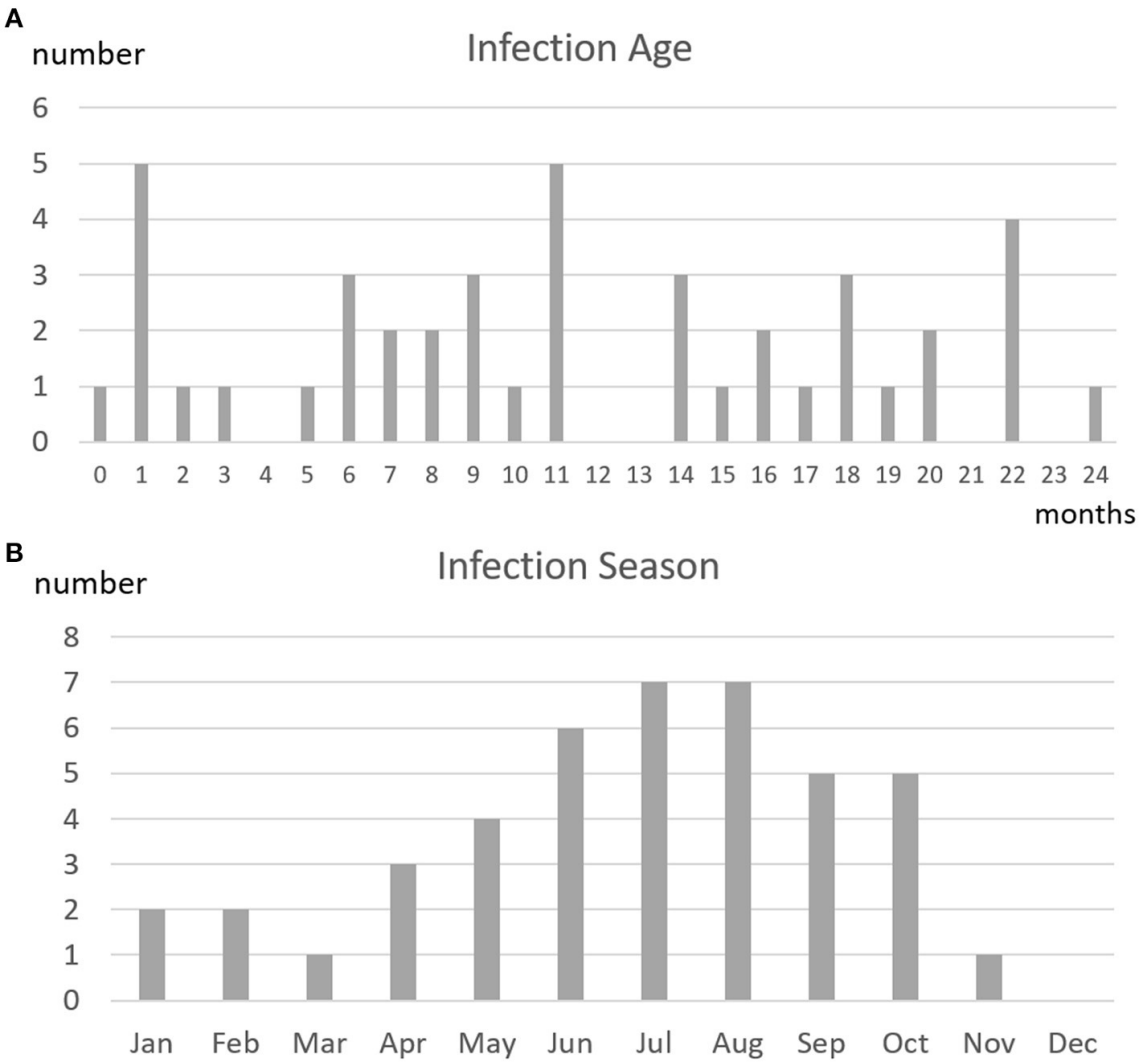
**(A)** Age at infection and **(B)** season of infection in 42 patients with CHD treated according to the subtropical guidelines on palivizumab prophylaxis who had 43 episodes of RSV-related hospitalizations. The distribution of ages at infection did not identify a high-risk age group. The graph showing the times of occurrence of RSV infections did not reveal RSV seasonality.

Figure 3A shows the 42 patients with CHD stratified by age at RSV-related hospitalization and Figure 3B shows the patients stratified by month of hospitalization. Of the total of 43 RSV-related hospitalizations, 25 infections occurred in patients aged ≤12 months and 18 infections occurred in patients aged 13–24 months.

Regarding the seasonality of RSV infections, the numbers of infections were highest in July and August, and were lowest from December to March. Nevertheless, RSV infections occurred throughout almost the entire year.

## Discussion

In this study, we reported the following findings: (1) most of the RSV infections occurring in patients with CHD who were treated based on the subtropical guidelines for palivizumab prophylaxis could be accounted for by the fact that the infected children no longer satisfied the criteria of the protocol for palivizumab prophylaxis, and not because of the ineffectiveness of palivizumab; (2) CHD patients with associated anomalies, especially those with genetic syndromes or lung/airway abnormalities, had increased risk for RSV-related hospitalizations, compared with patients without associated anomalies. The prophylaxis guidelines for patients with associated anomalies might require further adjustments by providing extra doses of palivizumab in those with genetic or lung/airway abnormalities.

### Effectiveness of Palivizumab Prophylaxis

Our previous multicenter study found that palivizumab prophylaxis administered according to the subtropical protocol decreased the RSV-related hospitalization rate of patients with cyanotic and hs-acyanotic CHD aged < 1 year by 49% (12). In our current study, none of the patients administered palivizumab prophylaxis died of RSV infection. In addition, following the subtropical guideline was economical, because the mean number of palivizumab injections was only 3.3 per patient. These results are consistent with the prophylactic effect of 5 injections of palivizumab administered during the RSV season by Feltes et al. Their multicenter study was performed in countries located in temperate zones and Western countries (4, 14).

In our current study of palivizumab prophylaxis, the difference between the rates of RSV-related hospitalization for patients aged < 1 year and patients aged > 1 and <2 years was not significant. This result contrasts with previous findings before the era of palivizumab prophylaxis that CHD patients with or without hemodynamically significant CHD with RSV infections who were aged < 2 years (1, 15) showed notably elevated hospitalization rates. Our results support the conclusion that palivizumab prophylaxis reduces RSV-related hospitalization rates in children with hsCHD aged < 1 year. The RSV hospitalization rate for unprophylaxed children 1–2 years of age was also relatively low (2.2%).

Although palivizumab prophylaxis is effective against RSV infections, some patients with CHD are still hospitalized for RSV infections. Our analysis of the relationship between RSV infection and palivizumab prophylaxis found that most (69%) of RSV-related hospitalizations could be accounted for by the fact that the study patients no longer satisfied the criteria of the subtropical guidelines for the use of palivizumab prophylaxis (hemodynamic condition improved in some children after surgical correction and other children were older than 1 year of age). The actual rate of failure of palivizumab prophylaxis used in children is low. To reduce RSV-related hospitalization rates further, the identification of high-risk children and revision of the guidelines for palivizumab prophylaxis that includes additional doses of palivizumab for high-risk patients may be of paramount importance.

### Impact of Associated Abnormalities

CHD is well-known to be associated with various comorbidities. Our previous study found that 4.8% of children with cyanotic CHD and 6.1% of children with hs-acyanotic CHD had associated chromosome anomalies (11). In children with a specific condition such as an extreme form of tetralogy of Fallot, the incidence of associated chromosome anomalies has been found to be even higher (16). The rate of associated airway anomalies was also especially elevated in patients with CHD associated with CATCH 22 syndrome (17). Additionally, the rate of neurological sequelae after CHD surgery was not uncommon. Whether associated abnormalities increase the risk of RSV-related hospitalization in CHD patients has not yet been studied (18, 19). Our current study found that with the current subtropical guidelines on palivizumab prophylaxis, the difference between the RSV-related hospitalization rates of children with cyanotic vs. acyanotic CHD was not significant. However, the rate of RSV-related hospitalization, was significantly higher for patients with than for patients without associated abnormalities (18–20).

Down syndrome has been found to be a risk factor for RSV-related hospitalization and severe RSV disease, independent of its association with CHD (21, 22). The Kids' Inpatient Database in the United States of America shows that the RSV-related hospitalization rate was decreased in patients with CHD and chronic lung disease in the palivizumab prophylaxis era (18). Patients with Down syndrome and congenital airway anomalies had the highest RSV-related hospitalization rate during the era of palivizumab prophylaxis. The study by Thorburn et al. found that children with severe RSV infections and pre-existing diseases such as chromosomal anomalies, cardiac lesions, neuromuscular disease, chronic lung disorders, and airway abnormalities had increased risk of mortality (23). In addition, children with 2 or more pre-existing conditions and RSV had a 4.3-fold increase in the risk of mortality. Our current study also found that children with hsCHD and comorbidities such as genetic syndromes and lung/airway abnormalities and RSV infections had increased risk of hospitalization and severe disease. Taken together the findings of these studies stress the important role of associated abnormalities in CHD patients with RSV infections.

The prematurity, especially those with chronic lung disease, was a notorious risk of RSV related hospitalization. It was traditionally the highest risk of RSV infection in the pre-palivizumab era (1). However, in the palivizumab prophylaxis era, the RSV hospitalization rate decreased dramatically in the patients with prematurity and chronic lung disease (18, 23). In our country, the palivizumab prophylaxis was reimbursed by National Health Insurance for prematurity < 32 weeks since 2010, which also decrease the RSV infection risk in the prematurity profoundly (24). In the present study, because of the small number of patients with prematurity, we didn't find association of RSV infection with prematurity. Therefore, we didn't stratify the patients according to the gestational age.

Our current study found that although patients with associated anomalies had a higher risk of RSV hospitalizations than patients without associated anomalies, they had received the similar doses of palivizumab, namely 3.3 doses per patient, which is lower than the 5 doses recommended by the AAP guidelines. We also found that patients with associated genetic syndromes and airway abnormalities received similar doses of palivizumab as the patients without associated genetic syndromes and airway abnormalities, although the former patients carried a higher risk of RSV infection. Revision of the subtropical protocol to give extra doses of palivizumab might be necessary for these subgroups of patients with CHD.

In the recent studies, the risk of RSV related hospitalization for hsCHD remains high in the second year of life (25, 26). Several studies have suggested to provide further palivizumab prophylaxis up to 2 years in these hsCHD (27, 28). However, the cost and benefit need to be justified. In the present study, we identify the persistent high-risk group, i.e., with associated genetic or lung/airway abnormalities, after maximal 6 doses of palivizumab prophylaxis in hsCHD. This finding provides evidence that selected patients with certain associated abnormalities may need further prophylaxis for RSV infection up to 2 years of age.

### Study Limitation

The study has several limitations that need to be addressed. First, the study was conducted in a single center which limits its generalizability to settings with similar protocols for RSV prophylaxis in children with hsCHD. The large, assembled cohort, however with an adequate follow-up period lends credence to the results of our study. Second, polymerase chain reaction was not performed in clinical practice which may have underestimated the true incidence of RSV infection, but the RSV detection methods utilized in our study are congruent with current RSV diagnostics reported in the literature. Third, all the patients enrolled in this prospective, descriptive study received prophylaxis. The absence of a control arm makes it difficult to ascertain the magnitude of the effect of prophylaxis on RSV-related hospitalizations in the sub-groups, but the comparative analysis indicates significantly higher hospitalization and intubation rates in hsCHD children with associated anomalies and higher ICU admission rates in those with genetic syndromes.

## Conclusion

Children with abnormalities, especially genetic syndromes and lung/airway problems associated with CHD are at high risk for RSV-related hospitalization. Our current subtropical guidelines for palivizumab prophylaxis in patients with hsCHD, should be revised to include the results of this study. Additional doses of palivizumab for prophylaxis may be necessary.

## Data Availability Statement

The original contributions presented in the study are included in the article/Supplementary Material, further inquiries can be directed to the corresponding author/s.

## Ethics Statement

The studies involving human participants were reviewed and approved by Institutional Review Board of National Taiwan University Hospital. Written informed consent to participate in this study was provided by the participants' legal guardian/next of kin.

## Author Contributions

S-NC: conceptualized and designed the study, coordinated and collected the data, wrote the first draft of the manuscript, analyzed the initial study, and reviewed and revised and final proved the manuscript. C-CW, M-TL, C-AC, C-WL, Y-CH, J-MW, and M-HW: designed the study, coordinated and collected the data, critically revised the initial manuscript, reviewed and final proved the manuscript. J-KW: conceptualized and designed the study, analyzed the study, coordinated and supervised data collection, critically revised the initial manuscript, reviewed and revised and final proved the manuscript. All authors contributed to the article and approved the submitted version.

## Funding

This study was supported by Taiwan Society of Pediatric Cardiology, Grant Number TSPC-202001.

## Conflict of Interest

The authors declare that the research was conducted in the absence of any commercial or financial relationships that could be construed as a potential conflict of interest.

## Publisher's Note

All claims expressed in this article are solely those of the authors and do not necessarily represent those of their affiliated organizations, or those of the publisher, the editors and the reviewers. Any product that may be evaluated in this article, or claim that may be made by its manufacturer, is not guaranteed or endorsed by the publisher.

## Supplementary Material

Supplementary Figure 1 The relationship of RSV-related hospitalization and palivizumab prophylaxis status in our CHD cohort.

Supplementary Table 1 Number and percentages of patients with congenital heart disease who were administered palivizumab doses (*N* = 772).

Supplementary Table 2 Mean numbers of doses of palivizumab administered to patients with CHD according to their associated abnormalities. All comparisons showed no statistically significant difference.
