# Supplementary figures and images for: Reappraisal of the Subtropical Guidelines on Palivizumab Prophylaxis in Congenital Heart Disease
Source: Front Pediatr. 2022 Jan 5;9:756787. doi: 10.3389/fped.2021.756787 (PMC8767946; doi:10.3389/fped.2021.756787)

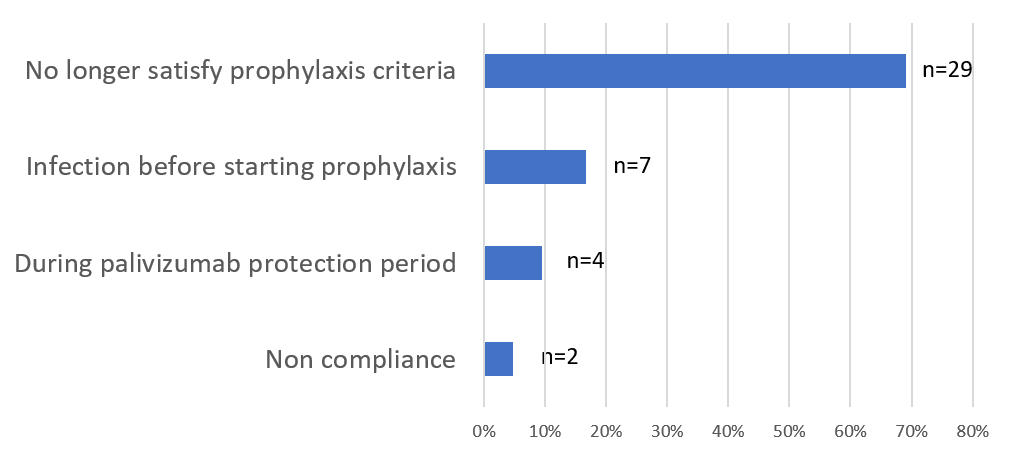

Supplement: Supplementary Figure 1 — The relationship of RSV-related hospitalization and palivizumab prophylaxis status in our CHD cohort. [file Image_1.TIF]
