# Supplementary material for: Reappraisal of the Subtropical Guidelines on Palivizumab Prophylaxis in Congenital Heart Disease
Source: Front Pediatr. 2022 Jan 5;9:756787. doi: 10.3389/fped.2021.756787 (PMC8767946; doi:10.3389/fped.2021.756787)
Supplement: Supplementary Table 1 — Number and percentages of patients with congenital heart disease who were administered palivizumab doses (N = 772). [file Data_Sheet_1.docx]

Supplementary Table 1. Number and percentages of patients with congenital heart disease who were administered palivizumab doses (N = 772)

| Number of palivizumab doses | 1 | 2 | 3 | 4 | 5 | 6 |
| --- | --- | --- | --- | --- | --- | --- |
| Number of patients | 269 | 100 | 65 | 44 | 66 | 228 |
| Percentage of patients | 35% | 13% | 8.4% | 5.7% | 8.5% | 30% |

Supplementary Table 2. Mean numbers of doses of palivizumab administered to patients with CHD according to their associated abnormalities. All comparisons showed no statistically significant difference.

|  | Mean doses of palivizumab |
| --- | --- |
| With associated abnormalities  Without | 3.4±2.1 3.3±2.1 |
| With genetic syndromes  Without | 3.3±2.1 3.3±2.1 |
| With airway/lung abnormalities  Without | 3.2±2.  3.3±2.1 |
| With neurological abnormalities  Without | 3.4±2.2 3.3±2.1 |
